# Inter-Individual Variability in the Adaptive Responses to Endurance and Sprint Interval Training: A Randomized Crossover Study

**DOI:** 10.1371/journal.pone.0167790

**Published:** 2016-12-09

**Authors:** Jacob T. Bonafiglia, Mario P. Rotundo, Jonathan P. Whittall, Trisha D. Scribbans, Ryan B. Graham, Brendon J. Gurd

**Affiliations:** 1 School of Kinesiology and Health Studies, Queen’s University, Kingston, Ontario, Canada; 2 School of Human Kinetics, University of Ottawa, Ottawa, Ontario, Canada; Universidad de las Palmas de Gran Canaria, SPAIN

## Abstract

The current study examined the adaptive response to both endurance (END) and sprint interval training (SIT) in a group of twenty-one recreationally active adults. All participants completed three weeks (four days/ week) of both END (30 minutes at ~65% VO_2_peak work rate (WR) and SIT (eight, 20-second intervals at ~170% VO_2_peak WR separated by 10 seconds of active rest) following a randomized crossover study design with a three-month washout period between training interventions. While a main effect of training was observed for VO_2_peak, lactate threshold, and submaximal heart rate (HR), considerable variability was observed in the individual responses to both END and SIT. No significant positive relationships were observed between END and SIT for individual changes in any variable. Non-responses were determined using two times the typical error (TE) of measurement for VO_2_peak (0.107 L/min), lactate threshold (15.7 W), and submaximal HR (10.7bpm). Non-responders in VO_2_peak, lactate threshold, and submaximal HR were observed following both END and SIT, however, the individual patterns of response differed following END and SIT. Interestingly, all individuals responded in at least one variable when exposed to both END and SIT. These results suggest that the individual response to exercise training is highly variable following different training protocols and that the incidence of non-response to exercise training may be reduced by changing the training stimulus for non-responders to three weeks of END or SIT.

## Introduction

Considerable heterogeneity exists in the individual response in peak oxygen uptake (VO_2_peak) following exercise training [1–3]. Specifically, VO_2_peak can increase [2,4], decrease [5], or remain unchanged [6,7] following structured endurance training (END). Similarly, inter-individual variability in training responses have also been observed following supra-maximal sprint interval training (SIT) [8,9]. While variability in training responses has been demonstrated following both END and SIT, it is currently unknown whether individuals who fail to respond following one type of exercise training might respond to a different training stimulus (i.e. different exercise volume, intensity and metabolic demand).

While END and SIT differ substantially in exercise volume, intensity, and metabolic demand, at the group level they induce strikingly similar adaptations in VO_2_peak [10,11], lactate threshold [12,13], and muscle oxidative potential [14–16]. Interestingly, limited evidence demonstrating that central adaptations following training may differ between END and SIT [17,18], supports the potential that the mechanisms underlying similar adaptations in VO_2_peak may differ following END and SIT. Further, individual variability in both peripheral [19,20] and central [17] adaptations following training have been observed. Together these results suggest that both central and peripheral adaptations may vary in an individual following END or SIT, supporting the hypothesis that an individual who fails to respond following END may respond following SIT (and *vis versa*).

Therefore, in order to determine if individuals respond differently to END and SIT, the present study compared individual responses following three weeks of both END and SIT utilizing a randomized crossover study design with a three-month washout period between training interventions. Individual changes in VO_2_peak, lactate threshold, and submaximal heart rate (HR) were compared and the incidence of response and non-response for all variables were classified using typical error (TE), an index of measurement error that considers both biological and technical variability [21]. We hypothesized that individual responses to END would not necessarily reflect responses to SIT (and *vis versa*), potentially due to differences in central and peripheral adaptations.

## Methods

Twenty-one healthy recreationally active (self-reported < three hours of physical activity per week) men (n = 9) and women (n = 12) volunteered to participate in the study. Each participant attended a preliminary screening session where they were briefed on the study, provided informed consent, and had their height and weight recorded. Participants were not previously trained in cycling and were not involved in a training program at the start of the study. Participants were informed to maintain their regular physical activity and nutritional habits throughout the duration of the study. All experimental procedures performed on human participants were approved by the Health Sciences Human Research Ethics board at Queen’s University. Verbal and written explanation of the experimental protocol and associated risks was provided to all participants prior to obtaining written informed consent.

### Experimental Design

**Fig 1:**
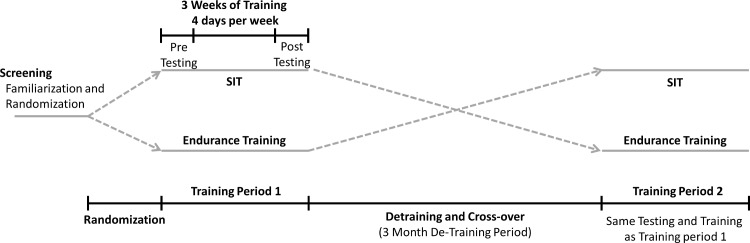
Overview of experimental protocol.

The current study utilized a randomized crossover design (Fig 1) where participants completed two, three-week training interventions separated by a three-month wash-out period during which participants were instructed to return to their pre-study levels of physical activity. Physiological testing occurred in the week preceding, and the week following each three-week training intervention. All physiological testing and training for both experiments was performed on a Monark Ergomedic 874 E stationary ergometer (Vansbro, Sweden). Eight additional participants completed a supplemental experiment to determine typical error for all variables. All participants were asked to refrain from alcohol and caffeine 12 hours before, and nutritional supplements and exercise 24 hours before all physiological testing.

### Physiological Testing

In the week preceding (pre) and the week following (post) training, participants reported to the lab on three separate occasions, separated by 24–48 hours. During each visit participants completed a VO_2_peak incremental ramp test to volitional exhaustion as described previously [22]. Briefly, following a 20 minute warm-up of four alternating five minute periods of load-less and 80W pedalling at 80 RPM, work rate was increased by 25W per minute until volitional exhaustion. Gas exchange and heart rate (HR) were collected throughout each ramp test using a metabolic cart (Moxus AEI Technologies, Pittsburgh, PA) and Polar HR monitors (Polar Team2 Pro, Kempele, Finland). VO_2_peak was calculated for each test as the highest 30 second average VO_2_ value, whereas submaximal HR was calculated for each test as the 30 second average HR value during the third stage of the ramp protocol (~156 W). Final pre- and post-training VO_2_peak and submaximal HR were determined by averaging the three values obtained during each testing period. RPM was collected continuously throughout each test and peak aerobic power (WR_peak_) was calculated using the average WR from the last 30 seconds of the test, whereas the WR at VO_2_peak was calculated using the average WR during the same 30 seconds used to calculate VO_2_peak.

### Lactate Threshold

Fingertip capillary blood (~20 uL) was sampled at rest (baseline) and within the last 10 s of each successive one-minute stage during the first VO_2_peak ramp test of pre- and post-testing test using a Lactate Scout + (EFK Diagnostics, Magdeberg, Germany) as done previously [13]. Lactate threshold was determined as the first recorded work rate (WR) where lactate was >4 mmol/L [23,24], often referred to as the onset of blood lactate accumulation at 4 mmol/L [25–27].

### Training Interventions

Training consisted of two, three week training periods separated by ~three months. During each training period participants were instructed to either cycle for 30 minutes at ~65% of WR at VO_2_peak (END) or perform eight, 20-second intervals at ~170% of WR at VO_2_peak, separated by 10 seconds of rest (SIT). Both training interventions required participants to train four times per week and the order of training was counterbalanced such that 12 (six males; six females) participants completed END first. All training sessions were preceded by a one-minute loadless warm-up. Participants were instructed to maintain a cadence of 80RPM and received verbal encouragement throughout all training sessions. HR was collected during training, at three minute intervals (END) and at the end of each interval (SIT), using Polar HR monitors (Polar Team2 Pro, Kempele, Finland). Ratings of perceived exertion (RPE) were collected immediately following each training session using a 6–20 Borg Scale [28]. HR and RPE were averaged over all training sessions to determine training HR and RPE for END and SIT.

### Determination of Typical Error

In order to determine typical error (TE) for VO_2_peak, lactate threshold and submaximal HR, a supplemental experiment involving eight recreationally active participants (four males; four females, age, 21±1 yrs; BMI, 21±2 kg/m^2^; VO_2_peak, 44±6 mL/kg/min) reported to the lab on two separate occasions separated by at least a week as described previously [9]. On each visit to the lab participants performed identical incremental ramp tests to volitional fatigue as described above. VO_2_peak, lactate threshold and submaximal HR were determined for each test as described above and the resulting values were utilized to calculate TE.

Typical error (TE) of measurement was calculated for VO_2_peak, lactate threshold, and submaximal HR as described previously [21] utilizing the following equation:

$$TE={SD}_{diff}/\sqrt{2}$$

Where *SD*_*diff*_ is the variance (standard deviation) of the difference scores observed between the 2 repeats of each test. A non-responder for VO_2_peak, lactate threshold, or submaximal HR was defined as an individual who failed to demonstrate an increase or decrease that was greater than two times the TE away from zero. A change beyond two times the TE means there is high probability (i.e. 12 to 1 odds) that this response is a true physiological adaptation beyond what might be expected to result from technical and/or biological variability [21].

### Statistical Analysis

Data are expressed as means and standard deviation. To ensure efficacy of the washout period baseline and response measures of VO_2_peak, lactate threshold, and submaximal HR between training period one and two were compared using unpaired t-tests as described previously [29]. Effects of training protocol (END vs. SIT) and time (Pre vs. Post) for all variables were examined using a two-way, repeated measures ANOVA. Any significant main effects or interactions were subsequently analyzed using a Bonferroni post hoc test where appropriate. Unpaired t-tests were also used to assess differences in the training response for all variables between males and females following END and SIT separately, and to determine if responses following END or SIT differed between training periods. A simple linear regression was used to determine the relationship between baseline variables between training period one and two and between the magnitude of response between END and SIT. Differences in training HR and RPE between END and SIT were assessed using paired t-tests and simple linear regressions were used to determine if these variables were related to the magnitude of physiological responses following training. A McNemar’s test was used to determine whether END and SIT elicited similar rates of response for VO_2_peak, lactate threshold, and submaximal HR. Statistical significance was accepted at *p* < 0.05.

## Results

Attendance at training sessions was 100% and all data reported are solely from those participants that completed the full study protocol. Three participants dropped out of the study following pre-training testing in the first training period and were not included in final analysis. Average HR during and RPE immediately following SIT (HR: 172.8 ± 7.8 bpm; RPE: 17.9 ± 0.9, mean ± SD) was significantly higher (*p* < 0.05) than END (HR: 166.2 ± 13.7 bpm; RPE: 15.5 ± 1.4, mean ± SD). Interestingly, RPE reported immediately following each SIT session was significantly related with the magnitude of change in VO_2_peak induced by SIT (r = 0.5, *p* < 0.05). Baseline measures, and the magnitude of response for all variables for training periods one and two are presented in Table 1. Unpaired t-tests revealed no differences between baseline measures for VO_2_peak (*p* = 0.62), lactate threshold (*p* = 0.12), and submaximal HR (*p* = 0.86) between training periods one and two. No differences were observed in the magnitude of response between training periods one and two for VO_2_peak (*p* = 0.20), lactate threshold (*p* = 0.55), or submaximal HR (*p* = 0.62). Additionally, there was no difference in the mean END or SIT response for any variable between training periods. Further, baseline measures for training periods one and two were significantly related for VO_2_peak (r = 0.94, *p* < 0.05), lactate threshold (r = 0.82, *p* < 0.05), and submaximal HR (r = 0.82, *p* < 0.05).

**Table 1:** Pre-training and magnitude of response for training periods 1 and 2 for all participants.

	Training Period One		Training Period Two	
	Pre-training	Response	Pre-training	Response
VO_2_peak (L/min)	3.0 ± 0.9	+0.05 ± 0.2	2.9 ± 0.9	+0.15 ± 0.3
VO_2_peak (mL/kg/min)	42.7 ± 6.4	+0.7 ± 3.2	41.2 ± 6.9	+2.2 ± 3.2
Lactate Threshold (W)	165.9 ± 43.2	+20.2 ± 18.3	190.7 ± 51.4	+14.7 ± 34.9
HR_submax_ (bpm)	153.9 ± 24.7	-5.8 ± 7.8	152.6 ± 21.5	-4.1 ± 12.8

Values are means ± standard deviation.

**Fig 2:**
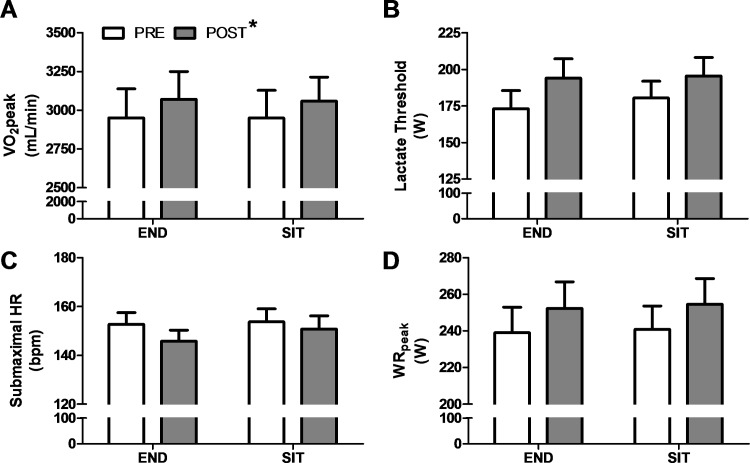
Group responses following 3 weeks of END and SIT. Group responses for VO_2_peak (A), lactate threshold (B), submaximal HR (C), and WR_peak_ (D). *Significant main effect of training, *p* < 0.05.

**Fig 3:**
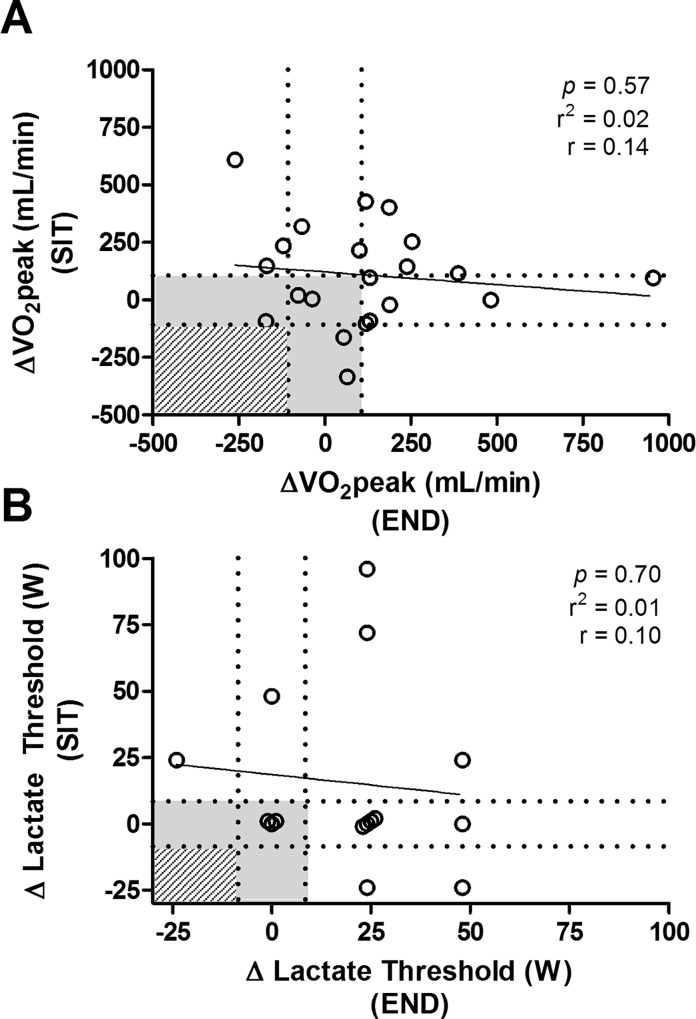
Correlations of individual responses following 3 weeks of END and SIT. Relationship between individual responses in VO_2_peak (A) and lactate threshold (B). Dashed lines represent the typical error cut-offs. Individuals falling within the shaded area failed to improve either VO2peak or lactate threshold following both END and SIT, while the hashed area represents an adverse response following both training protocols.

Participant characteristics and pre- and post-training values for END and SIT are presented in Table 2. A main effect of training (*p* < 0.05) was observed for VO_2_peak (Fig 2A), lactate threshold (Fig 2B), submaximal HR (Fig 2C), and WR_peak_ (Fig 2D). No condition (END vs. SIT) or interaction (condition x time) effects were observed for any variable examined. While males had higher baseline VO_2_peak, lactate threshold, submaximal HR, and WR_peak_ than females (*p* < 0.05), there were no statistical differences in the magnitude of training responses between sexes (Table 2). No significant relationships were observed between END and SIT for individual changes in VO_2_peak (r = 0.14, *p* = 0.57; Fig 3A), lactate threshold (r = 0.10, *p* = 0.70; Fig 3B), or submaximal HR (r = 0.17, *p* = 0.46). Baseline VO_2_peak did not predict changes in VO_2_peak following END (r = 0.28, *p* = 0.22) but was negatively related with the change in VO_2_peak induced by SIT (r = -0.59, *p* < 0.01). Baseline lactate threshold, and submaximal HR were not related with training-induced changes following either END (lactate threshold: r = 0.0, *p* = 1.0; HR: r = 0.37, *p* = 0.10) or SIT (lactate threshold: r = 0.29, *p* = 0.23; HR: r = 0.11, *p* = 0.63).

**Table 2:** Participant characteristics and group responses to END and SIT.

	END					
	Pre			Post		
	Males (n = 9)	Females (n = 12)	Total (n = 21)	Males (n = 9)	Females (n = 12)	Total (n = 21)
Age (yrs)	20.4 ± 1.2	19.9 ± 1.2	20.3 ± 0.9	-	-	-
Height (cm) †	181 ± 6	165 ± 7	172 ± 12	-	-	-
Body mass (kg) †	81.9 ± 10.3	62.0 ± 11.6	70.0 ± 14.7	82.0 ± 10.9	61.2 ± 11.5	69.5 ± 15.6
VO_2_peak (L/min) †	3.7 ± 0.5	2.4 ± 0.5	3.0 ± 0.9	3.8 ± 0.5	2.5 ± 0.5	3.1 ± 0.9*
VO_2_peak (mL/kg/min) †	46.0 ± 3.9	39.3 ± 6.7	42.2 ± 6.4	47.3 ± 5.4	41.4 ± 6.2	43.9 ± 6.4*
Lactate Threshold (W) †	209 ± 38	149 ± 40	175 ± 47	233 ± 40	171 ± 46	199 ± 51*
WR_peak_ (W) †	296 ± 42	196 ± 39	238 ± 64	309 ± 58	210 ± 32	252 ± 66*
HR_submax_ (bpm) †	135 ± 11	166 ± 19	152 ± 22.	129 ± 8	159 ± 18	146 ± 21*
	SIT					
	Pre			Post		
	Males (n = 9)	Females (n = 12)	Total (n = 21)	Males (n = 9)	Females (n = 12)	Total (n = 21)
Age (yrs)	20.4 ± 1.2	19.9 ± 1.2	20.3 ± 0.9	-	-	-
Height (cm)	181 ± 6	165 ± 7	172 ± 12	-	-	-
Body mass (kg) †	82.8 ± 11.6	62.2 ± 12.4	70.4 ± 15.6	83.1 ± 11.2	61.7 ± 11.5	70.3 ± 15.6
VO_2_peak (L/min) †	3.7 ± 0.6	2.4 ± 0.5	3.0 ± 0.9	3.7 ± 0.5	2.6 ± 0.4	3.1 ± 0.9*
VO_2_peak (mL/kg/min) †	45.0 ± 9.3	39.2 ± 5.5	41.7 ± 6.9	44.7 ± 5.5	41.6 ± 5.4	42.9 ± 5.5*
Lactate Threshold (W) †	215 ± 36	154 ± 40	180 ± 47	230 ± 33.3	165 ± 41	192 ± 47*
WR_peak_ (W) †	292 ± 45	202 ± 30	241 ± 57	314 ± 46	210 ± 32	255 ± 63*
HR_submax_ (bpm) †	133 ± 13	169 ± 19	155 ± 24	129 ± 9	167 ± 20	151 ± 25*

Values are means ± standard deviation. WR_peak,_ peak aerobic power; HR_submax_, submaximal heart rate.

†Significant baseline difference between males and females, *p* < 0.05.

*Main effect of training, *p* < 0.05.

**Fig 4:**
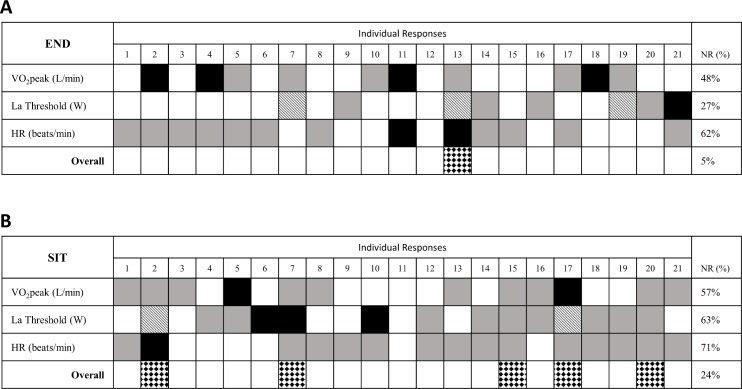
Individual patterns of response following three weeks of training. Positive responses (white boxes), non-responses (grey boxes) and adverse responses (black boxes) are shown for all participants across all variables following END (A) and SIT (B). A dashed box indicates that data was unavailable for a given variable. Individuals who failed to improve any variables for either END or SIT, “Overall non-responders” are indicated by diamond filled boxes. The percentage of participants demonstrating a non-response (NR; including both non- and adverse responses) for each variable, and overall, is also provided.

Unpaired t-tests revealed that the baseline characteristics of the participants used in the ancillary TE study did not statistically differ from the participants in the present study for all variables in Table 2. Two times TE was 0.107 L/min for VO_2_peak, 157 W for lactate threshold, and 10.0 bpm for submaximal HR. Individual patterns of response and rates of non-response for VO_2_peak, lactate threshold, and submaximal HR following both END and SIT are presented in Fig 4. Following training six non-responders were observed where an individual participant failed to improve in one measured variable following either END or SIT; however, in all cases these non-responders improved at least one variable following training utilizing the other exercise protocol. McNemar’s tests did not reveal significant differences in the incidence of response for VO_2_peak (*p* = 0.6), lactate threshold (*p* = 0.1), and submaximal HR (*p* = 0.6) between END and SIT.

## Discussion

The current study examined individual responses in VO_2_peak, lactate threshold and submaximal exercise heart rate (HR) following three weeks of both END and SIT. Carryover effects from training period one to two were absent and the magnitude of responses between training periods was not different for any variable. These data highlight the effective implementation of our randomized cross-over study design [29]. In summary, the current study demonstrates inter-individual variability in the training responses to END and SIT and suggests that individual patterns of response are dependent on the training protocol utilized.

The major novel findings of the current study are that: 1) while END and SIT increased VO_2_peak, lactate threshold and submaximal HR at the group level with no differences observed between protocols, improvements within a given individual following END did not predict the improvement observed following SIT (and *vice versa)*, 2) individual patterns of response were observed following both END and SIT, however these patterns varied within individuals between END and SIT, and 3) while our analysis revealed non-responses for one or more variables within most participants, we failed to observe a global non-response to END and SIT in any individual.

### Similar Group Responses in the Initial Adaptations to END and SIT

At the group level, END protocols are effective at increasing VO_2_peak and lactate threshold [30], while SIT protocols at supra-maximal intensities also improve VO_2_peak [31] and lactate threshold [12,13]. Consistent with these results and previous work form our lab utilizing the same protocols [16], a main effect of training was observed in the current study for VO_2_peak, lactate threshold, and submaximal HR. Also consistent with previous studies comparing END and SIT [14–16] no differences were observed between protocols for the magnitude of response at the group level. While the primary purpose of present study was not to determine if the group responses to END and SIT differ, the observation of similar group responses to END and SIT may suggest that a larger sample size is required to attain statistical power in order to detect potential interaction effects between training protocols.

### Individual Variability in Responsiveness to END and SIT

While variability in the individual responses to END is established [2,6,7,19,32,33], we recently demonstrated similar variability in response to the SIT protocol utilized in the present study [9]. The major novel finding of the current study is our demonstration of variability in the individual responses following different training protocols (END and SIT). Specifically, our results demonstrated that exercise protocols which differ in intensity, time, and metabolic demand, like END and SIT, can induce different adaptive responses in VO_2_peak, lactate threshold and submaximal HR within a given individual. These findings confirm the hypothesis that individuals who are not sensitive to a given exercise protocol may experience adaptation if exposed to a different protocol [5], potentially due to different sensitivities to training volume [7] and/or intensity [32]. While the mechanisms determining individual variability in sensitivities to differing training protocols are unknown, genetic predispositions [34] may be responsible for variance in the capacity of central [17] and peripheral [14,35] adaptations to training. A similar disassociation between individual changes in VO_2_peak has previously been observed following END and resistance training [33], but to our knowledge we are the first to demonstrate inter-individual variability in the response to two protocols known to induce equivalent improvements in aerobic capacity at the group level. Importantly, while the current data suggests that individuals may respond favorably to a change in training stimulus, we cannot rule out the possibility that the different individual responses observed between END and SIT were a result of simply training twice at two different times (i.e. it is possible that an individual completing END twice may not demonstrate identical responses), differences in external physical activity between training periods and/or changes in nutritional habits caused by different training protocols (i.e. END vs. SIT), or training at different times of the year (i.e. fall vs. winter). Additionally, the present study only examined individual variability in the initial response to training (i.e. the response to three weeks of training), and it remains possible that individual differences observed following three weeks of END and SIT may not persist following longer training periods. Thus, while our results support the consideration of multiple training protocols when attempting to optimize individual exercise prescription [36], there remains very little data, and much future work still needed, before we fully understand inter-individual responsiveness to different training protocols.

Consistent with previous observations of heterogeneity in the individual response to END [7,32,33,37] and SIT [8,9] we have also observed significant rates of non-response following both END and SIT in the current study (Fig 4). The present finding that END and SIT elicited similar rates of non-response for VO_2_peak, lactate threshold, and submaximal HR agrees with previous observations that END and SIT Importantly, while inter-individual variability in the response to training has been repeatedly demonstrated [7,33,37], attempts to quantify individuals as responders or non-responders are relatively recent [5–9,32,38]. In the current study, the use of two times the typical error (TE) to identify responders and non-responders [21] may have led to higher incidences of non-responses than previously reported [6–8,32]. However, despite the use of this conservative method of identifying responders, we have observed a subset of adverse responders to VO_2_peak, lactate threshold, and submaximal HR following both END and SIT that is consistent with previous observations of adverse responses to exercise for a variety of cardiovascular risk factors [5]. Interestingly, a non- or adverse response to VO_2_peak, lactate threshold, or submaximal HR following one training protocol did not preclude a positive response following the other training protocol. Recently, several reports have recommended that before individuals are classified as responders or non-responders, it is important to determine if variability in the individual responses within the experimental condition are greater than within-subject variation [39–41]. While we were unable to conduct this analysis due to our current study lacking a time-matched control group, it is important that future studies examining rates of response/non-response to exercise training consider the recently recommended approach to performing these analyses [39–41]. This the limitation aside, the current study adds to a growing body of literature that identifies a portion of the population that either does not respond, or responds adversely to exercise training and suggests that these non-/adverse-responders may respond more positively to different training protocols.

### Mechanisms Underlying Individual Variability to END and SIT

Despite marked differences in the physiological stress they impose, a single bout of END or SIT elicits analogous molecular responses in skeletal muscle [16], leading to similar peripheral adaptations including changes in fibre-type distribution [16], increased skeletal muscle oxidative capacity [14–16,42] and resting muscle glycogen content [14–16]. Interestingly, the central adaptations elicited by END or SIT are inconsistent [17,18], however, only central adaptations associated with six weeks of END prevails as independent predictors of the VO_2_peak responses [43]. Few studies have compared both central and peripheral adaptations to multiple training protocols and significant differences in training duration, frequency, and volume limits the ability to compare and interpret findings from different studies [43,44]. While recent research has elucidated mechanisms that primarily explain the adaptive responses to training [43], future research is needed to determine if variability in the mechanisms that underlie changes in exercise capacity/performance explain individual response variability following training.

At the individual level, heterogeneity in both central [17] and peripheral adaptations are present following END [19,20] and SIT [14], which suggests that variability in individual responses to END and SIT may be due in part to individual variance in the magnitude of peripheral and central adaptations. Why variance in central and/or peripheral adaptations may exist within an individual following different training protocols is currently unknown, however, evidence from the HERITAGE study suggests that much of this variability may results from a genetic predisposition to a specific type of training stimulus [2]. Interestingly, recent evidence has found associations between several genetic markers and individual training responses [34,45,46], however, while these findings are a step towards optimizing individual exercise prescriptions [34] whether genetic signatures exist that may predict which type of training an individual is most likely to respond to is unknown. This remains an interesting and important area for future investigation.

### Individual Patterns of Response

Following END and SIT we observed individual patterns of response, where improvements in VO_2_peak were not necessarily associated with improvements in lactate threshold or submaximal HR (Fig 4). The existence of individual patterns of response is consistent with previous studies demonstrating that non-responders in VO_2_peak can be responders to other variables associated with END [6,19] and SIT [8,9]. An additional novel finding of the present study is that individual patterns of response were different following END and SIT. This variability in individual patterns of response meant that even though several individuals failed to improve any variable following either END or SIT, no “global non-responders” (i.e. individuals that failed to improve following either protocol) were observed. These results further support the consideration of multiple training protocols when prescribing exercise, and raise the possibility that an individual who does not appear to be responding to an initial exercise prescription may respond more favourably if an alternative mode of training is prescribed. As continuing the training stimulus beyond initial exposure (four weeks) reduces the incidence of non-response in VO_2_peak [32], whether switching training protocols after initial exposure or extending the amount of training prescription is equally effective at diminishing non-responses remains an area for future research.

## Conclusion

The current study assessed individual responses in VO_2_peak, lactate threshold, and submaximal exercise heart rate (HR) following three weeks of both END and SIT. While training elicited significant improvements in all variables at the group level, considerable heterogeneity was observed in the individual responses including a number of non-/adverse-responders. Further, individual patterns of response were not related across END and SIT and appear to be training protocol dependent. All participants demonstrated a positive response in at least one variable following the completion of both END and SIT suggesting that the existence of true non-responders to exercise training is unlikely and that different training protocols should be considered when optimizing individual exercise prescription.

## Supporting Information

S1 Table Raw data used for all tables and figures. (XLSX)

## References

[1] Kohrt WM, Malley MT, Coggan AR, Spina RJ, Ogawa T, Ehsani AA, et al Effects of gender, age, and fitness level on response of VO2max to training in 60–71 yr olds. J Appl Physiol [Internet]. 1991 11 [cited 2016 Feb 28];71(5):2004–11. Available from: http://jap.physiology.org/content/71/5/2004.abstract 1761503 10.1152/jappl.1991.71.5.2004

[2] Bouchard C, Rankinen T. Individual differences in response to regular physical activity. Med Sci Sports Exerc. 2001;33(6 Suppl):S446–51; discussion S452–3. 11427769 10.1097/00005768-200106001-00013

[3] Skinner JS, Jaskólski a, Jaskólska a, Krasnoff J, Gagnon J, Leon a S, et al Age, sex, race, initial fitness, and response to training: the HERITAGE Family Study. J Appl Physiol. 2001;90(5):1770–6. 11299267 10.1152/jappl.2001.90.5.1770

[4] Karavirta L, Häkkinen K, Kauhanen A, Arija-Blázquez A, Sillanpää E, Rinkinen N, et al Individual responses to combined endurance and strength training in older adults. Med Sci Sports Exerc. 2011;43(3):484–90. 10.1249/MSS.0b013e3181f1bf0d 20689460

[5] Bouchard C, Blair SN, Church TS, Earnest CP, Hagberg JM, Häkkinen K, et al Adverse metabolic response to regular exercise: Is it a rare or common occurrence? PLoS One. 2012;7(5). 10.1371/journal.pone.0037887 PMC3364277 22666405

[6] Scharhag-Rosenberger F, Walitzek S, Kindermann W, Meyer T. Differences in adaptations to 1 year of aerobic endurance training: Individual patterns of nonresponse. Scand J Med Sci Sport. 2012;22(1):113–8. 10.1111/j.1600-0838.2010.01139.x 20561283

[7] Sisson SB, Katzmarzyk PT, Earnest CP, Bouchard C, Blair SN, Church TS. Volume of exercise and fitness nonresponse in sedentary, postmenopausal women. Med Sci Sports Exerc. 2009;41(3):539–45. 10.1249/MSS.0b013e3181896c4e 19204597 PMC2669311

[8] Astorino TA, Schubert MM. Individual responses to completion of short-term and chronic interval training: A retrospective study. PLoS One. 2014;9(5). 10.1371/journal.pone.0097638 PMC4029621 24847797

[9] Gurd BJ, Giles MD, Bonafiglia JT, Raleigh JP, Boyd JC, Ma JK, et al Incidence of nonresponse and individual patterns of response following sprint interval training. Appl Physiol Nutr Metab [Internet]. 2016;41(3):229–34. 10.1139/apnm-2015-0449 26854820

[10] Gist NH, Fedewa M V., Dishman RK, Cureton KJ. Sprint Interval Training Effects on Aerobic Capacity: A Systematic Review and Meta-Analysis. Sport Med [Internet]. 2014;44(2):269–79. Available from: http://link.springer.com/10.1007/s40279-013-0115-0 10.1007/s40279-013-0115-0 24129784

[11] Sloth M, Sloth D, Overgaard K, Dalgas U. Effects of sprint interval training on VO _2max_ and aerobic exercise performance: A systematic review and meta-analysis. Scand J Med Sci Sports [Internet]. 2013;23(6):e341–52. 10.1111/sms.12092 23889316

[12] Esfarjani F, Laursen PB. Manipulating high-intensity interval training: Effects on, the lactate threshold and 3000m running performance in moderately trained males. J Sci Med Sport [Internet]. 2007;10(1):27–35. Available from: http://linkinghub.elsevier.com/retrieve/pii/S1440244006001149 10.1016/j.jsams.2006.05.014 16876479

[13] Zelt JGE, Hankinson PB, Foster WS, Williams CB, Reynolds J, Garneys E, et al Reducing the volume of sprint interval training does not diminish maximal and submaximal performance gains in healthy men. Eur J Appl Physiol [Internet]. 2014; Available from: http://www.ncbi.nlm.nih.gov/pubmed/25091854 10.1007/s00421-014-2960-4 25091854

[14] Gibala MJ, Little JP, van Essen M, Wilkin GP, Burgomaster KA, Safdar A, et al Short-term sprint interval versus traditional endurance training: similar initial adaptations in human skeletal muscle and exercise performance. J Physiol. 2006;575(Pt 3):901–11. 10.1113/jphysiol.2006.112094 16825308 PMC1995688

[15] Burgomaster K a, Howarth KR, Phillips SM, Rakobowchuk M, Macdonald MJ, McGee SL, et al Similar metabolic adaptations during exercise after low volume sprint interval and traditional endurance training in humans. J Physiol. 2008;586(1):151–60. 10.1113/jphysiol.2007.142109 17991697 PMC2375551

[16] Scribbans TD, Edgett BA, Vorobej K, Mitchell AS, Joanisse SD, Matusiak JBL, et al Fibre-specific responses to endurance and low volume high intensity interval training: Striking similarities in acute and chronic adaptation. PLoS One. 2014;9(6). 10.1371/journal.pone.0098119 PMC4047011 24901767

[17] MacPherson REK, Hazell TJ, Olver TD, Paterson DH, Lemon PWR. Run sprint interval training improves aerobic performance but not maximal cardiac output. Med Sci Sports Exerc. 2011;43(1):115–22. 10.1249/MSS.0b013e3181e5eacd 20473222

[18] Matsuo T, Saotome K, Seino S, Shimojo N, Matsushita A, Iemitsu M, et al Effects of a low-volume aerobic-type interval exercise on $\dot{V}O$ 2max and cardiac mass. Med Sci Sports Exerc. 2014;46(1):42–50. 10.1249/MSS.0b013e3182a38da8 23846165

[19] Vollaard NBJ, Constantin-Teodosiu D, Fredriksson K, Rooyackers O, Jansson E, Greenhaff PL, et al Systematic analysis of adaptations in aerobic capacity and submaximal energy metabolism provides a unique insight into determinants of human aerobic performance. J Appl Physiol. 2009;106(5):1479–86. 10.1152/japplphysiol.91453.2008 19196912

[20] McPhee JS, Williams AG, Perez-Schindler J, Degens H, Baar K, Jones DA. Variability in the magnitude of response of metabolic enzymes reveals patterns of co-ordinated expression following endurance training in women. Exp Physiol. 2011;96(7):699–707. 10.1113/expphysiol.2011.057729 21571817

[21] Hopkins WG. Measures of reliability in sports medicine and science. Sports Med. 2000;30(1):1–15. 10907753 10.2165/00007256-200030010-00001

[22] Edgett BA, Foster WS, Hankinson PB, Simpson CA, Little JP, Graham RB, et al Dissociation of Increases in PGC-1α and Its Regulators from Exercise Intensity and Muscle Activation Following Acute Exercise. PLoS One [Internet]. 2013;8(8):e71623 Available from: http://dx.plos.org/10.1371/journal.pone.0071623 10.1371/journal.pone.0071623 23951207 PMC3741131

[23] Bishop D, Jenkins DG, Mackinnon LT. The relationship between plasma lactate parameters, Wpeak and 1-h cycling performance in women. Med Sci Sports Exerc [Internet]. 1998 8 [cited 2015 Nov 25];30(8):1270–5. Available from: http://www.ncbi.nlm.nih.gov/pubmed/9710868 9710868 10.1097/00005768-199808000-00014

[24] Heck H, Mader A, Hess G, Mucka S, Muller R, Hollmann W. Justification of the 4-mmol/l Lactate Threshold. Int J Sports Med. 1985;6(3):117–30. 10.1055/s-2008-1025824 4030186

[25] Bentley DJ. Incremental Exercise Test Design and Analysis. Sport Med. 2007;37(7):575–86. 10.2165/00007256-200737070-00002 17595153

[26] Svedahl K, MacIntosh BR. Anaerobic threshold: the concept and methods of measurement. Can J Appl Physiol. 2003;28(2):299–323. 12825337 10.1139/h03-023

[27] Faude O, Kindermann W, Meyer T. Lactate threshold concepts: How valid are they? Sport Med [Internet]. 2009;39(6):469–90. Available from: file:///C:/Users/mitch_000/Downloads/Faude-SportsMed-2009-SG-Fisiologia.pdf 10.2165/00007256-200939060-00003 19453206

[28] Borg GA V. Psychophysical bases of percieved exertion. Med Sci Sports Exerc. 1982;14(5):377–81. 7154893

[29] Wellek S, Blettner M. On the Proper Use of the Crossover Design in Clinical Trials. Dtsch Arztebl Int. 2012;109(15):276–81. 10.3238/arztebl.2012.0276 22567063 PMC3345345

[30] Jones AM, Carter H. The effect of endurance training on parameters of aerobic fitness. Sports Med. 2000;29(6):373–86. 10870864 10.2165/00007256-200029060-00001

[31] Bacon AP, Carter RE, Ogle EA, Joyner MJ. VO2max Trainability and High Intensity Interval Training in Humans: A Meta-Analysis. PLoS One. 2013;8(9). 10.1371/journal.pone.0073182 PMC3774727 24066036

[32] Ross R, de Lannoy L, Stotz PJ. Separate Effects of Intensity and Amount of Exercise on Interindividual Cardiorespiratory Fitness Response. Mayo Clin Proc [Internet]. Elsevier Inc; 2015;90(11):1–9. Available from: http://linkinghub.elsevier.com/retrieve/pii/S0025619615006400 26455890 10.1016/j.mayocp.2015.07.024

[33] Hautala AJ, Kiviniemi AM, Mäkikallio TH, Kinnunen H, Nissilä S, Huikuri H V., et al Individual differences in the responses to endurance and resistance training. Eur J Appl Physiol. 2006;96(5):535–42. 10.1007/s00421-005-0116-2 16369817

[34] Timmons JA, Knudsen S, Rankinen T, Koch LG, Jensen T, Keller P, et al Using molecular classification to predict gains in maximal aerobic capacity following endurance exercise training in humans programs Using molecular classification to predict gains in maximal aerobic capacity following endurance exercise training in human. J Appl Physiol. 2012; 10.1152/japplphysiol.01295.2009 PMC2886694 20133430

[35] Granata C, Oliveira RSF, Little JP, Renner K, Bishop DJ. Training intensity modulates changes in PGC-1 and p53 protein content and mitochondrial respiration, but not markers of mitochondrial content in human skeletal muscle. FASEB J [Internet]. 2015 10.1096/fj.15-276907 26572168

[36] Buford TW, Roberts MD, Church TS. Toward exercise as personalized medicine. Sport Med. 2013;43(3):157–65. 10.1007/s40279-013-0018-0 PMC3595541 23382011

[37] Bouchard C, An P, Rice T, Skinner JS, Wilmore JH, Gagnon J, et al Familial aggregation ofV˙o 2 max response to exercise training: results from the HERITAGE Family Study. J Appl Physiol [Internet]. 1999;87(3):1003–8. Available from: http://jap.physiology.org/content/87/3/1003.abstract 10484570 10.1152/jappl.1999.87.3.1003

[38] Wolpern AE, Burgos DJ, Janot JM, Dalleck LC. Is a threshold-based model a superior method to the relative percent concept for establishing individual exercise intensity? a randomized controlled trial. BMC Sports Sci Med Rehabil [Internet]. BMC Sports Science, Medicine and Rehabilitation; 2015;7(1):16 Available from: http://www.biomedcentral.com/2052-1847/7/16 26146564 10.1186/s13102-015-0011-z PMC4491229

[39] Hopkins WG. Individual responses made easy. J Appl Physiol [Internet]. 2015;118(12):1444–6. 10.1152/japplphysiol.00098.2015 25678695

[40] Hecksteden A, Kraushaar J, Scharhag-Rosenberger F, Theisen D, Senn S, Meyer T. Individual response to exercise training -a statistical perspective. J Appl Physiol. 2015;118:1450–9. 10.1152/japplphysiol.00714.2014 25663672

[41] Atkinson G, Batterham AM. True and false inter-individual differences in the physiological response to an intervention. Exp Physiol [Internet]. 2015;6:1–29. Available from: http://www.ncbi.nlm.nih.gov/pubmed/25823596 10.1113/EP085070 25823596

[42] Ma JK, Scribbans TD, Edgett BA, Boyd CJ, Simpson CA, Little JP, et al Extremely low-volume, high-intensity interval training improves exercise capacity and increases mitochondrial protein content in human skeletal muscle. Open J Mol Integr Physiol [Internet]. 2013;03(04):202–10. Available from: http://www.scirp.org/journal/PaperInformation.aspx?PaperID=39842&#abstract

[43] Montero D, Cathomen A, Jacobs RA, Flück D, de Leur J, Keiser S, et al Haematological rather than skeletal muscle adaptations contribute to the increase in peak oxygen uptake induced by moderate endurance training. J Physiol [Internet]. 2015;593(20):4677–88. 10.1113/JP270250 26282186 PMC4606528

[44] Jacobs RA, Flück D, Bonne TC, Bürgi S, Christensen PM, Toigo M, et al Improvements in exercise performance with high-intensity interval training coincide with an increase in skeletal muscle mitochondrial content and function. J Appl Physiol [Internet]. 2013;115(6):785–93. Available from: http://www.ncbi.nlm.nih.gov/pubmed/23788574 10.1152/japplphysiol.00445.2013 23788574

[45] Bouchard C, Sarzynski MA, Rice TK, Kraus WE, Church TS, Sung YJ, et al Genomic predictors of the maximal O_2_ uptake response to standardized exercise training programs. J Appl Physiol. 2011;110(5):1160–70. 10.1152/japplphysiol.00973.2010 21183627 PMC3098655

[46] He Z, Hu Y, Feng L, Li Y, Liu G, Xi Y, et al NRF-1 genotypes and endurance exercise capacity in young Chinese men. Br J Sports Med. 2008;42(5):361–6. 10.1136/bjsm.2007.042945 18184751

